# MiR-124 suppresses tumor growth and metastasis by targeting Foxq1 in nasopharyngeal carcinoma

**DOI:** 10.1186/1476-4598-13-186

**Published:** 2014-08-07

**Authors:** Xiao Hong Peng, Hao Ran Huang, Juan Lu, Xiong Liu, Fei Peng Zhao, Bao Zhang, Shao Xiong Lin, Lu Wang, Huai Hong Chen, Xia Xu, Fan Wang, Xiang Ping Li

**Affiliations:** Department of Otorhinolaryngology-Head and Neck Surgery, Nanfang Hospital, Southern Medical University, Guangzhou, Guangdong 510515 China; School of Public Health and Tropical Medicine, Southern Medical University, Guangzhou, 510515 China; Department of Otorhinolaryngology-Head and Neck Surgery, The First Affiliated Hospital of Shantou University Medical College, Shantou, Guangdong 515041 China

**Keywords:** MicroRNA-124, Tumor growth, Metastasis, Nasopharyngeal carcinoma, Foxq1

## Abstract

**Background:**

The molecular mechanisms underlying dysregulation of microRNAs have been documented in nasopharyngeal carcinoma (NPC). Our previous study demonstrated that plasma miR-124 was down-regulated in NPC using microarray analysis and quantitative PCR validation. Though growing studies showed that down-regulated miR-124 was closely related to tumourigenesis in various types of cancers, the role of miR-124 in NPC remains largely unknown.

**Methods:**

The expression level of miR-124 was evaluated in NPC cell lines and patient specimens using quantitative reverse transcription-PCR (Real-time qPCR). The clinicopathological significance of the resultant data was later analyzed. Then, we explored the role of miR-124 in NPC tumorigenesis by *in vitro* and *in vivo* experiments. Homo sapiens forkhead box Q1 (Foxq1) was confirmed as a novel direct target gene of miR-124 by the dual-luciferase assay and western bolt.

**Results:**

We found that miR-124 was commonly down-regulated in NPC specimens and NPC cell lines. The expression of miR-124 was inversely correlation with clinical stages and marked on T stages. Then, the ectopic expression of miR-124 dramatically inhibited cell proliferation, colony formation, migration and invasion *in vitro,* as well as tumor growth and metastasis *in vivo*. Furthermore, we identified Foxq1 as a novel direct target of miR-124. Functional studies showed that knockdown of Foxq1 inhibited cell growth, migration and invasion, whereas Foxq1 overexpression partially rescued the suppressive effect of miR-124 in NPC. In clinical specimens, Foxq1 was commonly up-regulated in NPC, and the level increased with clinical stages and T stages. Additionally, the level of Foxq1 was inversely correlated with miR-124.

**Conclusions:**

Our results demonstrate that miR-124 functions as a tumor-suppressive microRNA in NPC, and that its suppressive effects are mediated chiefly by repressing Foxq1 expression. MiR-124 could serve as an independent biomarker to identify patients with different clinical characteristics. Therefore, our findings provide valuable clues toward the understanding the of mechanisms of NPC pathogenesis and provide an opportunity to develop new effective clinical therapies in the future.

**Electronic supplementary material:**

The online version of this article (doi:10.1186/1476-4598-13-186) contains supplementary material, which is available to authorized users.

## Background

Nasopharyngeal carcinoma (NPC) is a non-lymphomatous squamous cell carcinoma derived from epithelial cells lining on the nasopharynx [1]. The characteristics are highly malignant local invasion and early distant metastasis, and the prevalence of NPC is low globally but high in southern China and Southeast Asia [2, 3]. The radiotherapy proves to be the most sensitive and effective treatment, but the average 5­year survival rate for NPC patients remains at 70% [1, 3–5]. During tumorigenesis and progression, multiple genetic and epigenetic abnormalities synergistically disrupt normal cell function, thus contributing to NPC pathogenesis [6–9]. Until now, the underlying mechanisms of NPC tumorigenesis are not completely understood. Therefore, further investigation is urgently needed.

MicroRNAs (miRs) are small non-coding RNAs which function as endogenous regulatory RNA molecules and modulate many physiological and pathological processes through down-regulating target genes [10, 11]. There is a large body of evidence indicating that abnormal miRNAs can function as oncogenes or tumor suppressors in tumor progression [12]. To date, multiple miRNAs have been shown to be dysregulated in NPC, such as miR-26a, miR-9, miR-10b, miR-144 and miR-214 which contributed to the development and progression of NPC [13–20]. Our previous study demonstrated for the first time that plasma miR-124 was down-regulated in NPC [21]. However, the role of miR-124 in NPC and the molecular mechanisms in which miR-124 exerts its functions remain largely unknown.

Human Foxq1 is first identified to encode a protein of 403 amino acids and belongs to the family of the Fox transcription factors (previously called HNF-3/ forkhead transcription factors) in 2001 [22]. The biological function of Foxq1 has been clearly identified in hair follicle differentiation [23, 24]. Previous studies have found that Foxq1 is widely expressed at the mRNA level in murine tissues, with particularly high expression levels in the bladder and stomach [25]. Recent several studies reported that Foxq1 was markedly overexpressed in colorectal cancer and glioma, which enhanced tumorigenicity and tumor growth *in vivo*[26, 27]. Furthermore, Foxq1 was also involved in epithelial-mesenchymal transition regulation by suppressing E-cadherin transcription, and associated with aggressive cancer phenotype [14, 15]. However, whether Foxq1 expression contributes to NPC development and progression is not clear.

In this study, we investigated the potential involvement of miR-124 in NPC. We examined the expression level of miR-124 in human NPC cells and tissues and tested its effects on cell growth, migration and invasion. In addition, we also investigated a potential role of miR-124 on NPC tumorigenesis and tumor metastasis in murine models. Finally, we explored the underlying mechanism of miR-124 functions in NPC. Our study will provide a better understanding of NPC pathogenesis.

## Results

### MiR-124 was down-regulated in NPC cell lines and clinical specimens and associated with advanced clinical stage

**Figure 1:**
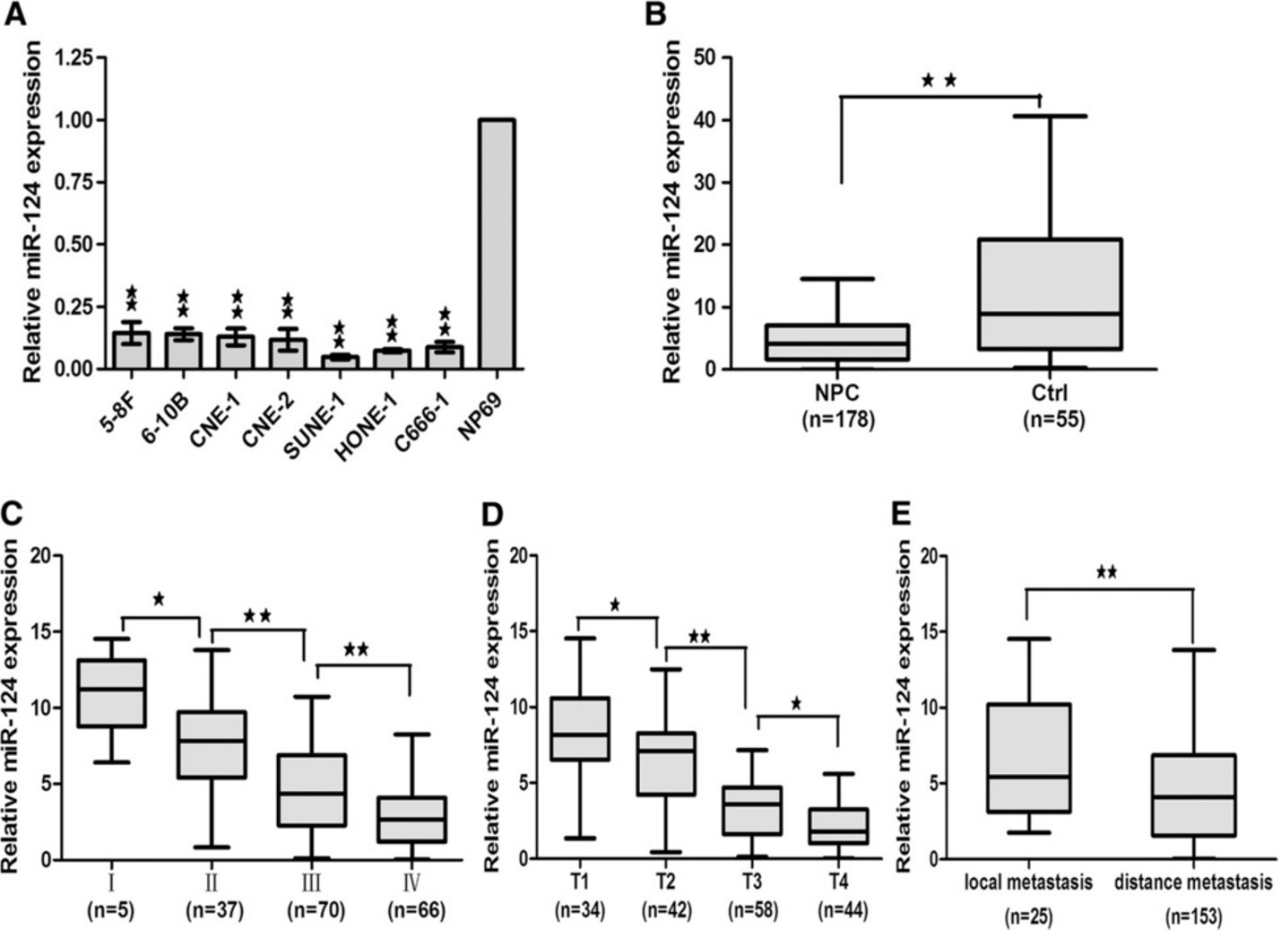
**The expression level of miR-124 was reduced in NPC cell lines and clinical specimens. A**, The expression of miR-124 was reduced in NPC cell lines. **B**, the average expression level of miR-124 in human NPC specimens compared with non-cancer biopsy samples. **C**, miR-124 expression was higher in stage I, whereas stages II-IV had lower levels. **D**, miR-124 expression was higher in stage T1, whereas stages T2-T4 had lower levels. **E**. The expression level of mir-124 in the distance metastasis was lower than in the local metastasis. Statistical analysis was performed using the nonparametric tests **(B)** and the one-way ANOVA **(C, D and** **E)**. *, P < 0.05, **, P < 0.01.

To study the expression level of miR-124 in NPC, a panel of NPC cell lines was first analyzed by Real-time PCR. Compared with the immortalized nasopharyngeal epithelial cell line NP69, the basal expression level of miR-124 was generally down regulated in 7 NPC cell lines (5-8 F, 6-10B, CNE1, CNE2, HONE-1, C666-1 and Sune-1) (Figure 1A).

We further tested the expression level of miR-124 in 178 primary NPC tissues and 55 non-cancer nasopharyngitis biopsy samples to analyze the clinicopathologic significance of miR-124. The relationship between the miR-124 expression level and clinicopathologic characteristics in NPC patients were summarized in Table 1. MiR-124 was not significantly associated with age and gender of the patients. Consistent with the result of the NPC cell lines, the average expression level of miR-124 was decreased in NPC tissue compared with non-cancer biopsy samples (Figure 1B). We found that miR-124 expression was higher in stage I, whereas stages II-IV had lower levels, showing a significant correlation of miR-124 with clinical stages (Figure 1C). Furthermore, we found that the miR-124 expression was higher in stage T1, whereas stages T2-T4 had lower levels, showing a significant correlation of miR-124 with T stage (Figure 1D). In addition, we found that the level of miR-124 was lower in distance metastasis tissues compared with local metastasis tissues (Figure 1E). Taken together, these data provided strong evidence that miR-124 expression was closely related to the progression and clinicopathologic features of NPC. On the basis of these results, we focused on miR-124 for further functional studies to evaluate its roles in NPC pathogenesis.

**Table 1:** **The relationship between miR-124 expression and clinicopathological parameters in 178 NPC patients**

Variable	No.	Median expression of miR-124		***P***
		High expression	Low expression	
Age, year				0.188
<45	95	46 (48.4%)	49 (51.6%)	
≥45	83	43 (51.8%)	40 (49.2%)	
Gender				0.749
Male	120	57 (47.5%)	63 (52.5%)	
Female	58	32 (55.1%)	26 (44.9%)	
T status				<0.001
T1	34	34 (100%)	0 (0%)	
T2	42	33 (78.6%)	9 (21.4%)	
T3	58	18 (31%)	40 (69%)	
T4	44	6 (13.6%)	38 (86.4%)	
N status				0.148
N0	16	10 (62.5%)	6 (37.5%)	
N1	80	44 (55%)	36 (45%)	
N2	60	27 (45%)	33 (55.0%)	
N3	22	8 (36.4%)	14 (63.6%)	
M status				0.114
M0	166	82 (50%)	84 (50%)	
M1	12	7 (58.0%)	5 (42.0%)	
Stage				
I	5	5 (100%)	0 (0%)	<0.001
II	37	33 (89.1%)	4 (10.8%)	
III	70	35 (50%)	35 (50%)	
IV	60	16 (26.7%)	44 (73.3%)	

### MiR-124 suppressed the proliferative, migratory and invasive capacities of NPC cells

**Figure 2:**
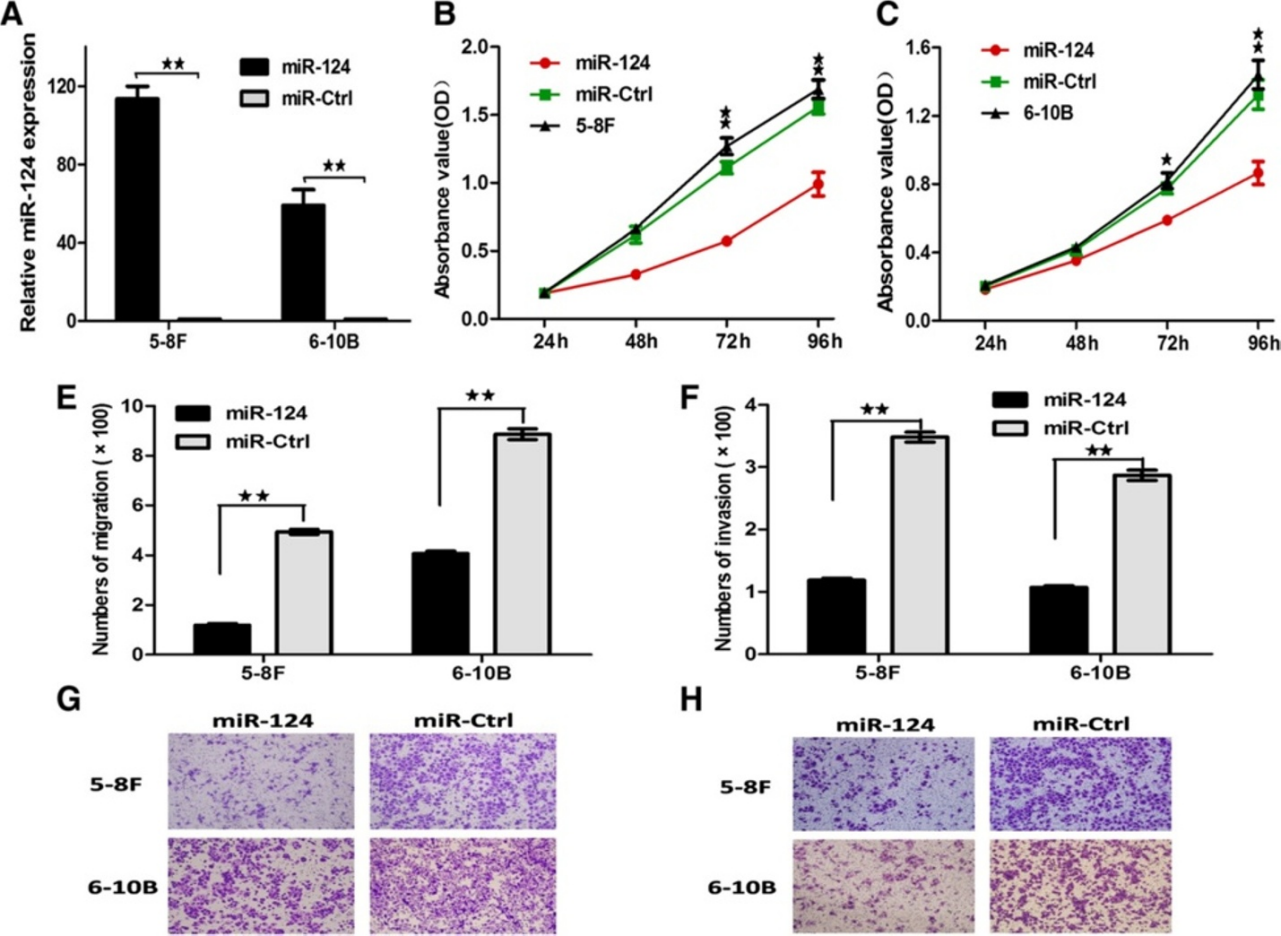
**The ectopic expression of miR-124 suppressed the proliferation, migratory and invasive capacity of NPC cell. A**, The expression levels of miR-124 in 5-8 F and 6-10b cell lines were verified after tranfected with miR-124 mimics and miR-Ctrl. **(B and** **C)**, Effect of miR-124 on cell proliferation was measured by CCK-8 assay in 5-8 F and 6-10b cell lines. **(E and** **F)**, The migrated and invasive cell numbers of NPC cells after transfected with miR-124 mimics and miR-Ctrl. **(G and** **H)**, The fields of migrated and invasive cells on the membrane (magnification × 100). Statistical analysis was performed using the t-tests. The data represent the mean values of three independent experiments. *, P < 0.05, **, P < 0.01.

**Figure 3:**
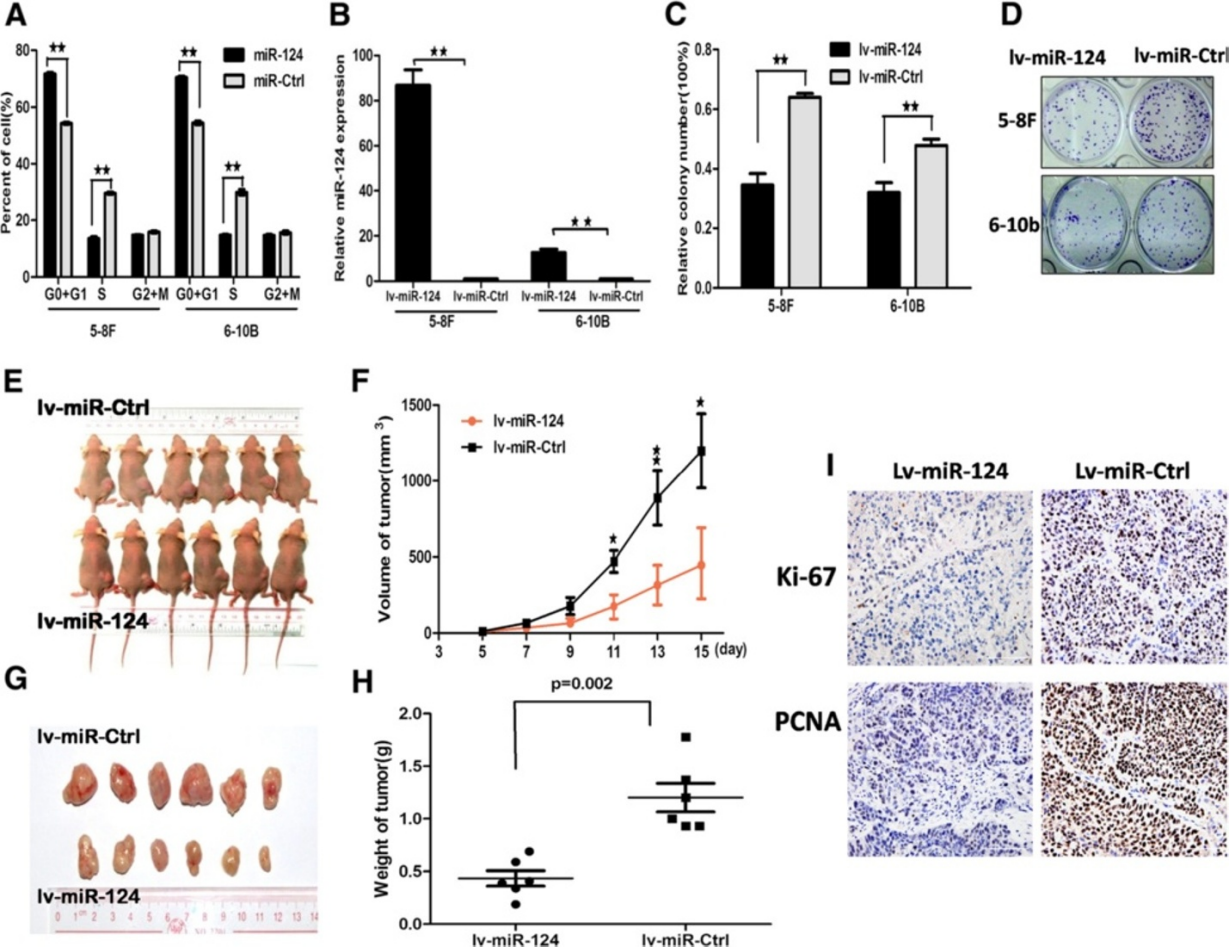
**The ectopic of miR-124 affected cell cycle in the NPC cell lines** ***in vitro*** **and suppressed cell growth** ***in vivo.*** **A**, The ectopic expression of miR-124 arrest G0 + G1 phases and decreased S phase in 5-8 F and 6-10b compared with miR-Ctrl. **B**, Stably expression of miR-124 cell lines were evaluated using real time qPCR. **C**, Colonies were evaluated and values were reported as the ratio between lv-miR-124-infected cells and lv-miR-Ctrl infected cells. **D**, Pictures of colony formation assay of lv-miR-124 infected 5-8 F and 6-10b cell lines. **(E and** **F)**, 5-8 F cells infected lv-miR-124 and lv-miR-Ctrl were injected subcutaneously into nude mice, at 15 days after implanted, lv-miR-124 infected 5-8 F cells produced smaller tumors than control cells. The “**F**” figure showed the growth curve of tumors volumes. Each data point represents of 6 mice. **G**, Picture showed the tumors after stripped. **H**, The weight of tumors compared with 5-8 F infected lv-miR-124 and lv-miR-Ctrl. **I**, Ki-67- and PCNA-stained sections of transplanted tumors formed after orthotopic transplantation. Scale bars, 100 μm. Statistical analysis was performed using the nonparametric tests. *, P < 0.05, **, P < 0.01.

To determine the role of miR-124, we transfected the NPC cell lines 5-8 F and 6-10B with miR-124 mimics and miR-Ctrl. The up-regulated expression of miR-124 was confirmed by Real-time PCR (Figure 2A). CCK-8 array was utilized to evaluate cell proliferative capacity. These results showed that ectopic expression of miR-124 could dramatically repress the cell growth of 5-8 F and 6-10B cells respectively (Figure 2B and C). Then, we tested the cell cycle distribution. The results demonstrated that ectopic expression of miR-124 arrest G0 + G1 phase and decreased S phase in 5-8 F and 6-10B cells compared with miR-Ctrl (Figure 3A). After analyzing the proliferation capacity of miR-124, we investigated the cell migration and invasion capacities. Migration and invasion arrays demonstrated that miR-124 overexpression inhibited cell migration and invasion compared with miR-Ctrl (Figure 2E, F, G and H).

To further investigate colony formation capacity, we used lentiviral vectors to stably up-regulate the expression level of miR-124 in 5-8 F and 6-10B cell lines (Figure 3B). Colony formation assay was performed to evaluate cell growth. Similar results indicated that miR-124 inhibited the colony formation ability in lv-miR-124/5-8 F and lv-miR-124/6-10B cells compared with lv-miR-Ctrl cells respectively (Figure 3C and D). Above all, these results supported that ectopic expression of miR-124 inhibited cell growth, colony formation, migration and invasion in NPC cell lines *in vitro*.

### MiR-124 inhibited tumor growth and metastasis *in vivo*

To determine whether miR-124 could affect tumor growth *in vivo*. Lv-miR-124/5-8 F cells and lv-miR-Ctrl cells were used for evaluating the effect of miR-124 overexpression on the growth of tumor xenografts. We implanted lv-miR-124/5-8 F or lv-miR-Ctrl/5-8 F cells subcutaneously in nude mice respectively (n = 6 per group). As shown in Figure 3E, lv-miR-124/5-8 F cells resulted in an approximately 2.68-fold decrease in tumor size relative to lv-miR-Ctrl/5-8 F cells after 15 days (Figure 3F, *P* < 0.05). After the tumors were dissected and weighed, the results of tumor weight were similar to results of tumor volume (Figure 3G and H). The expression levels of miR-124 were examined after the tumors were dissected (Additional file 1: Figure S1A). Thus, these results showed that miR-124 suppressed tumor proliferation capacity *in vivo*. We also showed that both the staining intensity and the number of hyperproliferative Ki-67^+^ and PCNA^+^ tumor cells were significantly decreased compared with control (Figure 3I and Additional file 2: Table S1; P < 0.05).

**Figure 4:**
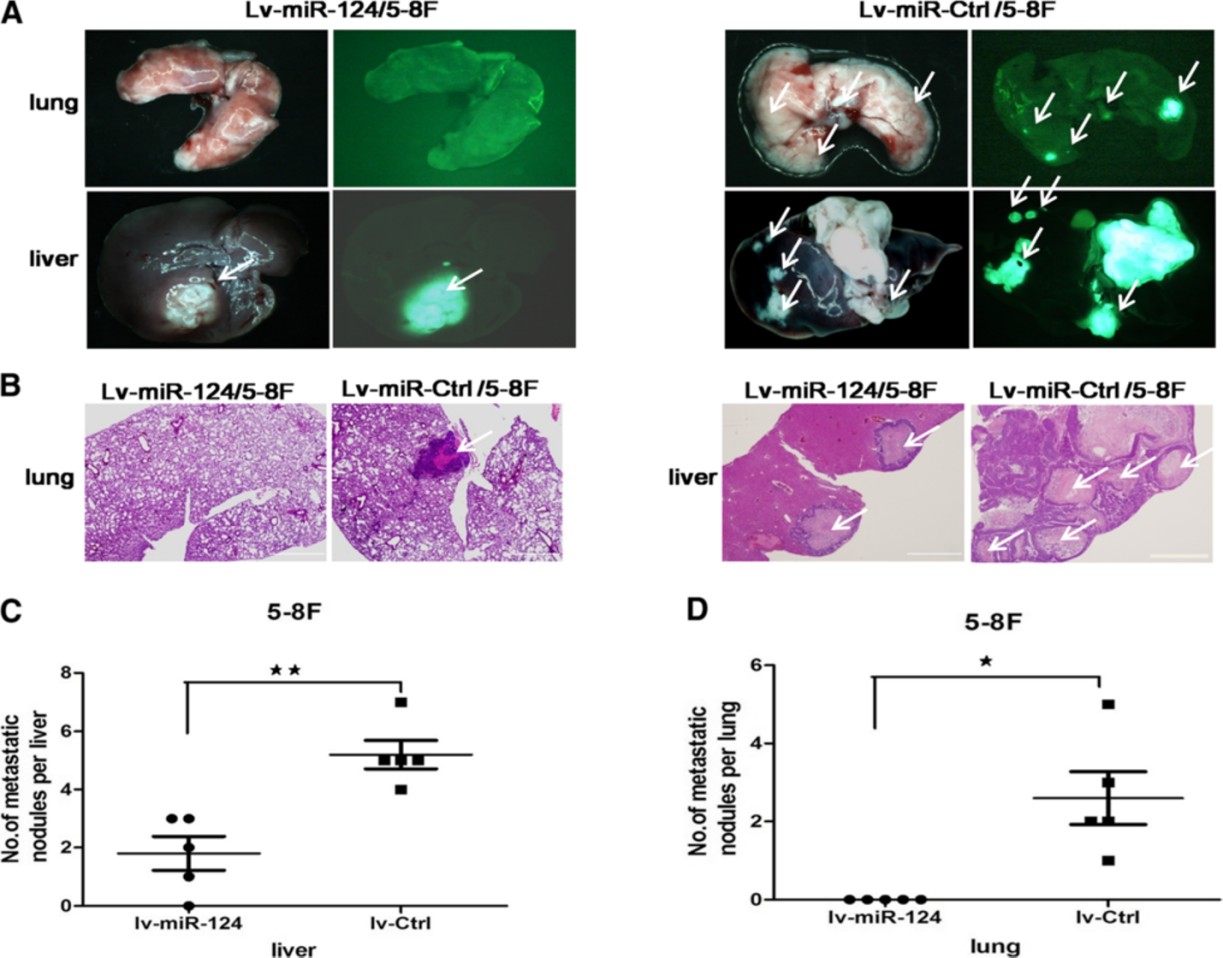
**MiR-124 suppressed the lung and liver metastases of NPC cells** ***in vivo*** **.** Groups of BALB/c nude mice were inoculated with lv-miR-124/5-8 F or lv-Ctrl/5-8 F cells. The liver and lung metastases were monitored by GFP-based fluorescence imaging on day 25 after inoculation (n = 5 per group). **A**, Lungs and livers were obtained from the mice of the lv-miR-124/5-8 F and lv-Ctrl/5-8 F groups. White arrows indicate liver and lung metastases. **B**, Representative H&E sections of liver and lung. Scale bars in lung, 200 μm; scale bars in liver, 2 mm. **(C and** **D)**, Numbers of the metastatic nodules in the liver and lungs after injected with lv-miR-124/5-8 F or lv-Ctrl/5-8 F cell. Statistical analysis was performed using the nonparametric tests. *, P < 0.05, **, P < 0.01.

To further investigate the metastatic effect of miR-124 *in vivo,* primary tumors were established by direct injection of lv-miR-124/5-8 F or lv-miR-Ctrl/5-8 F cells into the liver. Twenty-five days after transplantation, the mice were killed and the livers and lungs were dissected for macroscopic and microscopic histology. Livers and lungs of mice bearing miR-124-expressing 5-8 F tumors harbored statistically significantly fewer microscopic and macroscopic metastases than those of mice bearing mock-infected 5-8 F tumors (*P* < 0.05; Figure 4A-D). The expression levels of miR-124 were examined after the tumors were dissected from the livers (Additional file 1: Figure S1B).

### MiR-124 down-regulated the expression of Foxq1 by directly targeting its 3′-UTR

**Figure 5:**
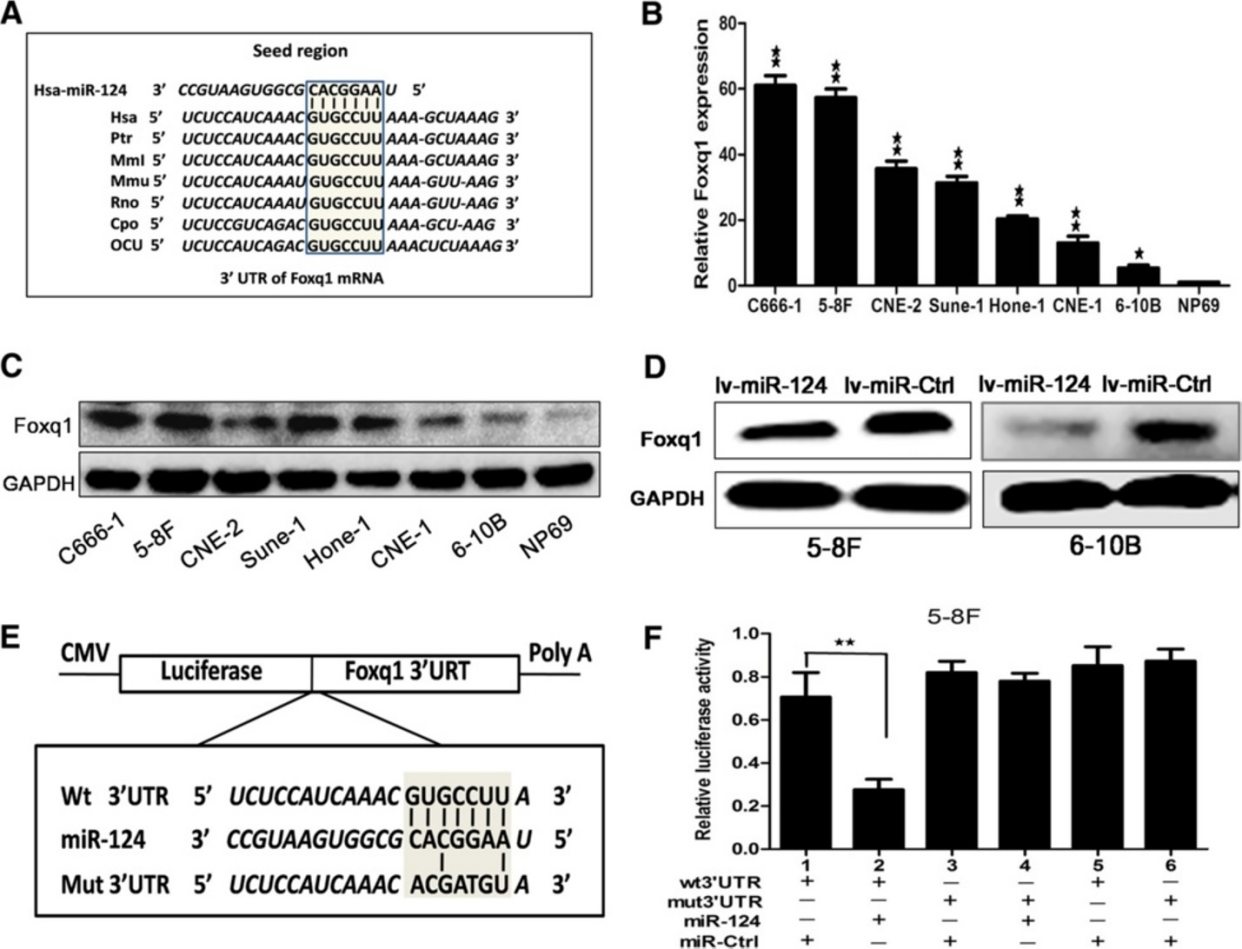
**The ectopic miR-124 induced the expression of Foxq1 by directly targeting its 3**′**-UTR. A**, Putative miR-124 binding site in the 3′-UTR region of Foxq1 and interspecies conservation of seed matching sequences (gray box). **(B** **and C)**, The gene expression of Foxq1 in NPC cells compared with NP69 cells by RT-qPCR and western bolt. **D**, The protein expression level of Foxq1 in lv-miR-124/5-8 F cell and lv-miR-124/6-10B cell compared with control. **E**, Diagram of wt 3′-UTR and mut 3′-UTR of Foxq1 contained reporter constructs. **F**, Luciferase reporter assays in 5-8 F cells, co-transfected of wt/mut 3′-UTR with miRNAs as indicated. Statistical analysis was performed using the t-tests. The data represent the mean values of three independent experiments. *, P < 0.05, **, P < 0.01.

To investigate the molecular mechanism for the proliferation, migration and invasion of suppression by miR-124 in NPC cells, we focused on 27 possible target genes of mir-124 by utilizing bioinformatic analysis. The selected target genes have been verified that could affect tumor growth or metastasis in the website (http://www.ncbi.nlm.nih.gov/nucleotide/). The selected targets were validated by RT-qPCR in lv-miR-124/5-8 F cells. The result of RT-qPCR showed that the expression level of Foxq1 decreased more significantly, compared with other 26 possible target genes (Additional file 3: Figure S2A). Finally, we selected Foxq1 gene as the target gene of miR-124. Foxq1 is highly conserved among different species, whose 3′-UTR of mRNA contained a complementary site for the seed region of miR-124 (Figure 5A). Real-time PCR and western blot was performed to detect the expression level of Foxq1, and the results showed that Foxq1 was up-regulated in 7 NPC cell lines compared with NP69 (Figure 5B and C). Then we analyzed the the mRNA and protein level of Foxq1 after transfected lv-miR-124 into 5-8 F and 6-10B cell lines. As shown in Figure 5D and Additional file 3: Figure S2B, Foxq1 was down-regulated after transfected lv-miR-124.

To further confirm that Foxq1 was a direct target of miR-124, dual-luciferase assay was performed. The target region sequence of Foxq1 3′-UTR (wt 3′-UTR) or the mutant sequence (mut 3′-UTR) was cloned into a luciferase reporter vector (Figure 5E). These recombinant vectors were co-transfected with miR-124 mimics or miR-Ctrl into 5-8 F cell line. The results showed that miR-124 could down-regulated the luciferase activity of the Foxq1 wt 3′-UTR construct (Figure 5F, lanes 1 and 2;P < 0.01). The activity of mut3′-UTR vector was unaffected by a simultaneous transfection with miR-124 (Figure 5F, lanes 3 and 4). Moreover, miR-Ctrl did not significantly affect the luciferase activity of either the wt or mut 3′-UTR construct (Figure 5F, lanes 5 and 6). The same results were obtained in HEK 293 T cell line (Additional file 3: Figure S2C). In summary, these results strongly suggested that miR-124 directly regulated Foxq1 in NPC cell lines.

### Overexpression of Foxq1 could partially rescue the suppression of miR-124

To explore the function of Foxq1 in NPC cell lines, Foxq1-siRNAs were transfected into 5-8 F cell lines. Real-time PCR was confirmed that the Foxq1 mRNA level expression was down-regulated compared with miR-Ctrl after transfected (Additional file 4: Figure S3A). CCK8 array showed that Foxq1 silencing inhibited the proliferation (Additional file 4: Figure S3B). Furthermore, migration and invasion arrays obtained the same results that knockdown of Foxq1 repressed the migration and invasion capacities of 5-8 F cells (Additional file 4: Figure S3C and D). According to these results, Foxq1 functioned as a potential oncogene in NPC cell lines.

**Figure 6:**
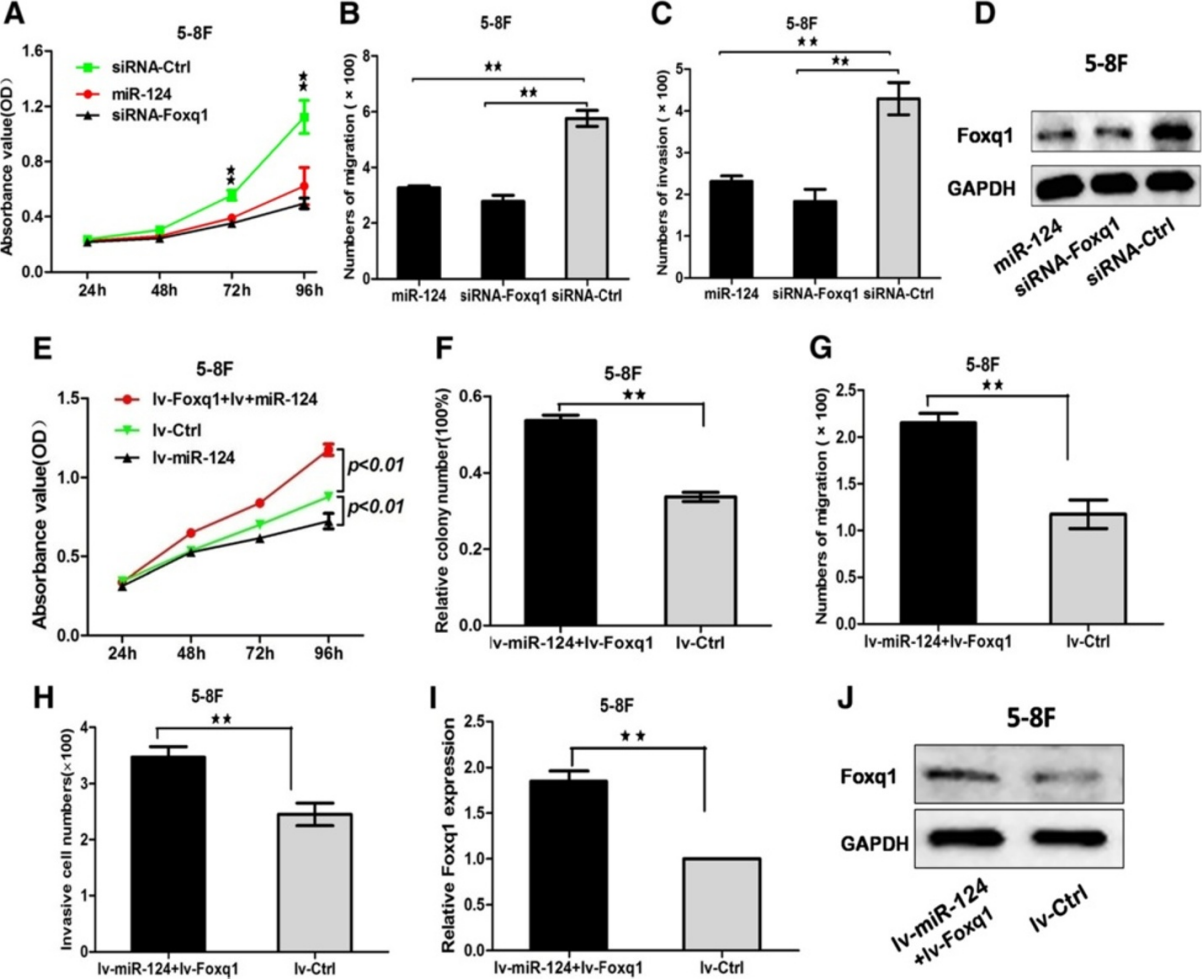
**The suppression of down-regulated of FOXQ1 was consistent with the suppression of the ectopic miR-124 and overexpression of FOXQ1 could rescue partially the suppression of miR-124. A**, 5-8 F cells were transfected with siRNA-Foxq1 or miR-124 mimics. Effect of siRNA-Foxq1 or miR-124 on cell proliferation was measured by CCK-8 assay in 5-8 F lines. **(B and** **C)**, The migrated and invasive cell numbers of NPC cells. **D**, The protein expression level of Foxq1 was detected after transfect with siRNA-Foxq1 or miR-124 mimics. **(E and** **F),** Effect of over-expression regulated Foxq1 in lv-miR-124/5-8 F cells on cell proliferation and tablet cloning ability were measured. **(G and** **H),** Effect of up-regulated Foxq1 in lv-miR-124/5-8 F cells on cell migration and invasion were test. **(I** **and J)**, Stable expression of Foxq1 in lv-miR-124/5-8 F cells (lv-Foxq1/lv-miR-124/5-8 F) was constructed. Statistical analysis was performed using the t-tests. The data represent the mean values of three independent experiments. **, P < 0.01.

To elucidate whether the suppressive effect of miR-124 was mediated by repression of Foxq1 in NPC cells, we performed gain-of-function and loss-of-function studies. First, we silenced Foxq1 to investigate whether the reduced expression of Foxq1 could mimic the suppressive effect of miR-124. 5-8 F cells were transfected with siRNA-Foxq1 or miR-124 mimics. The Foxq1 protein expression of siRNA-Foxq1/5-8F and miR-124/5-8F were down-regulated compare with siRNA-Ctrl/5-8F (Figure 6D). Then, we examined cell proliferative, migratory and invasive capacities. As shown in Figure 6A-C, Foxq1 knockdown led to significant suppressive effects, similar to those induced by miR-124 (*P* < 0.01). The similar results were obtained in 6-10B cell lines (Additional file 4: Figure S3E-G). Subsequently, we evaluated whether ectopic expression of Foxq1 could rescue the suppressive effect of miR-124. The lv-miR-124/5-8 F cell line after transfected lv-Foxq1 was named lv-miR-124 + lv-Foxq1/5-8 F. The expression level of Foxq1 was tested using Real-time PCR and Western bolt (Figure 6I and J). CCK8 array and colony-forming assay showed that Foxq1 could partially abrogate the effects mediated by miR-124 in lv-miR-124 + lv-Foxq1/5-8 F cells (Figure 6E and F). At the same time, the migration and invasion arrays showed that Foxq1 could partially restore the migration and invasion activity compared with the lv-Ctrl/5-8 F cells (Figure 6G and H). The similar results were obtained in lv-miR-124 + lv-Foxq1/6-10B cell lines (Additional file 5: Figure S4A-F). Therefore, we showed that Foxq1 could partially rescue the suppression of miR-124 in NPC cells.

### MiR-124 and Foxq1 were inversely correlated in NPC tissues

**Figure 7:**
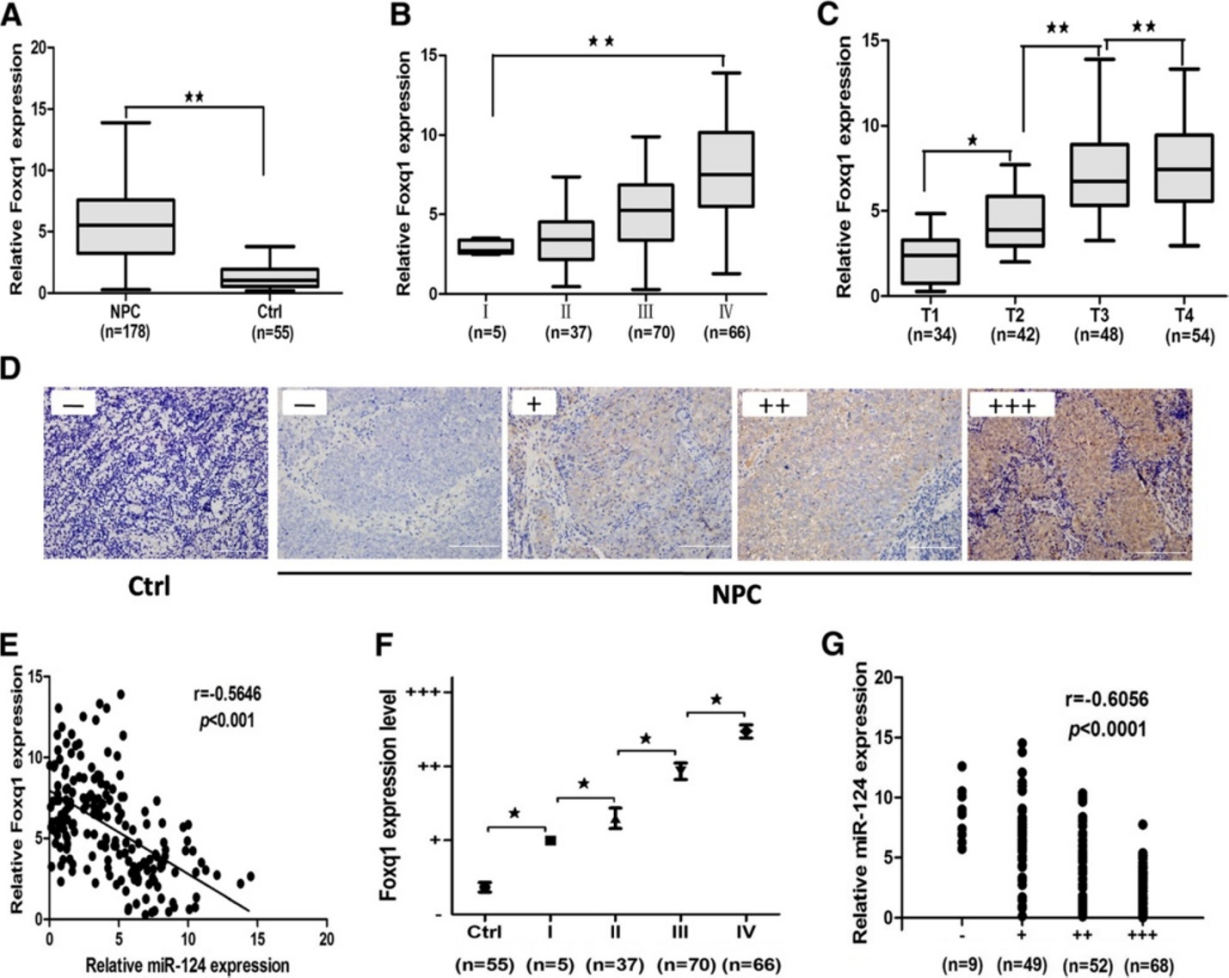
**Mir-124 and Foxq1 are inversely correlated in NPC tissues. A**, The average expression level of Foxq1 in human NPC specimens compared with non-cancer biopsy samples. **B**, The Foxq1 expression of clinicalIstage had lower expression than in the IVstage. **C**, Foxq1 expression was lower in stage T1, whereas stages T2-T4 had higher levels. **D**, Representative IHC for Foxq1 in non-cancer biopsy samples and NPC specimen with different clinical stages. **E**, In the mRNA levels, a significant inverse correlation was observed after correlated Foxq1 with the miR-124 expression levels in the 178 NPC specimens. **F**, Statistical quantification of the IHC scores of Foxq1 between non-cancer biopsy samples and NPC specimen with different clinical stages. Scale bars, 100 μm. **G**, A scatter diagram shows an inverse correlation between miR-124 and Foxq1 expression in the same set of NPC tissue (Spearman’s correlation analysis, r = -0.6056; *p <* 0.0001).Statistical analysis was performed using the nonparametric tests **(A)** and the one-way ANOVA **(B, C, E, F)**. *, P < 0.05, **, P < 0.01.

To further investigate the expression of Foxq1, 178 clinical human primary NPC tissues and 55 non-cancer nasopharyngitis biopsy samples were tested for analyzing the clinicopathologic significance. We found that the average expression level of Foxq1 was up-regulated in NPC tissue compared with non-cancer biopsy samples (Figure 7A). Further analysis found that the Foxq1 expression of clinical I stage was the lowest in the clinical stages (Figure 7B). In addition, we found that Foxq1 expression was lower in stage T1, whereas stages T2-T4 had higher levels, showing a significant correlation of Foxq1 with T stage (Figure 7C). We then correlated Foxq1 with the miR-124 expression levels in the same NPC specimens. As shown in Figure 7E, significant inverse correlation was observed when Foxq1 expression levels were plotted against miR-124 expression levels (2-tailed Spearman’s correlation, r = -0.5646; *p* < 0.001).

Immunohistochemistry (IHC) was also performed to detect Foxq1 expression in 178 clinical NPC specimens and 55 non-cancer nasopharyngitis biopsy samples. Foxq1 was found to be predominantly overexpressed in the cytoplasm and membranes of NPC cells and was less expressed in non-cancer nasopharyngitis biopsy samples (Figure 7D). Our data also showed that the Foxq1 expression was statistically higher than in non-cancer biopsy samples. We found that Foxq1 expression was lower in stage I, whereas stages II-IV had higher levels, showing a significant correlation of Foxq1 with clinical stages (P < 0.05, Figure 7F). Then we correlated Foxq1 with the miR-124 expression levels in the same NPC specimens. As shown in Figure 7G, a significant inverse correlation was observed when Foxq1 expression levels were plotted against miR-124 expression levels (2-tailed Spearman’s correlation, r = -0.6056; *p* < 0.0001).

## Discussion

In this report, we identified for the first time that miR-124 was markedly down-regulated in NPC cell lines and clinical specimens. The ectopic expression of miR-124 suppressed the proliferative, migratory and invasive capacities of NPC cells *in vitro*, and suppressed tumor growth and metastasis *in vivo*. Moreover, we found that the expression of Foxq1 protein was down-regulated after transfected lv-miR-124 in NPC cells by western blot. By using Dual-luciferase assay, Foxq1 was identified as a new direct and functional target of miR-124. Our results also showed that Foxq1 overexpression could rescue partially the suppressive effect of miR-124, and we found a negative correlation between miR-124 and Foxq1 expression in NPC tissues. These results supported that Foxq1 was a direct target gene of miR-124. Our study demonstrated that miR-124 acts as a novel proliferation and metastasis suppressor by targeting Foxq1 in NPC.

The ectopic expression of miR-124 is a frequently epigenetically silenced tumor-suppressive microRNA in various cancers [28–34]. The capability of cells to proliferate, migrate and invade is considered an important determinant in the process of tumorigenesis and progression. Many oncogenes and suppressor genes reportedly correlate with the course of cancer initiation and progression [28]. In this study, our results provided strong evidences that miR-124 inhibited the proliferation, migration and invasion of NPC cells *in vitro* and *in vivo*. These results were consistent with observations in breast cancer, hepatocellular carcinoma and glioblastoma [28–34]. The suppressive capability suggested that miR-124 functioned as a tumor-suppressive microRNA in NPC.

Emerging evidence indicates that miR-124 is abnormally expressed and has been implicated in several tumors. Until now, several studies have demonstrated that miR-124 was down-regulated and inversely correlated with clinical characteristic and prognosis in breast cancer and colorectal cancer [28, 35]. Our previous study demonstrated that plasma miR-124 was down-regulated in NPC. The present study is the first report to explore the expression of miR-124 in NPC tissues. We found that the expression level of miR-124 between NPC patient plasma and tissue decreased simultaneously. The reduced expression of miR-124 in NPC tissues was inversely correlated with clinical stages, T stages and marked the progression from locoregional tumors to metastatic tumors. Recent reports have showed that miRNAs which can be reliably isolated from human plasma are stable in circulation, and can be used as a diagnostic tool for early detection of NPC [11, 36]. More importantly, it is simple, effective, and non-invasive blood-based biomarker which predicts the clinical behaviour of NPC and monitored therapeutic response [21]. Compared with the results in the present study, the expression level of miR-124 between NPC patient plasma and tissue changed in the same direction. The reduced expression of miR-124 in NPC tissues was inversely correlated with clinical characteristic. For these reasons, miR-124 could serve as an independent biomarker marker to identify patients with clinical characteristic. However, further investigation is needed to confirm the prognostic value of miR-124 as an effective biomarker.

Foxq1 is a member of the FOX gene family. FOX genes are involved in embryonic development, cell cycle regulation, tissue-specific gene expression, cell signaling, and tumorigenesis [22, 37]. Recent studies have clearly showed that Foxq1 was implicated in tumor proliferation and metastasis in colorectal cancer, glioblastoma, breast cancer and hepatocellular carcinoma [26, 27, 38, 39]. Foxq1 was a prognostic marker for patients in gastric cancer and hepatocellular carcinoma [40, 41]. However, the role of Foxq1 is rarely known in NPC. Our results demonstrated for the first time that Foxq1 was overexpressed in NPC cell lines and NPC tissues, consistent with the published data [27, 39, 40, 42]. In NPC tissues, Foxq1 expression increased with clinical stage, T stage. Functional studies also confirmed that down-regulation of Foxq1 suppressed cell proliferation, migration and invasion *in vitro*. These results implied that Foxq1 was a potential oncogene in development and progression of NPC. Nonetheless, the molecular pathological mechanism of Foxq1 in NPC was still unknown. A report showed that Foxq1 may be a novel target of the Wnt pathway in solid tumors and Foxq1 expression was associated with genes as markers for proliferation (MKI67, TPX2, AURKA) and epithelial-mesenchymal transition (VIM, SNAI1, ZEB2, CDH2) [42]. Then, we tested the mRNA levels of these genes and found that these genes (MKI67, SNAI1 and ZEB2) were down-regulated after siRNA- Foxq1 transfection in NPC cells (data not shown). Further study is needed to clearly illustrate the tumorigenic mechanisms of Foxq1.

## Conclusions

This study identifies miR-124 as a growth suppressive miRNA in human NPC, at least, partly through repression of Foxq1. MiR-124 could serve as an independent biomarker marker to identify patients with clinical characteristic. Although miRNA-based therapeutics is still in their infancy, our findings on miR-124 are encouraging and suggest that this miRNA could be a potential target for the treatment of NPC in future.

## Methods

### Clinical specimens

Tumors biopsy specimens (n = 178) and non-cancer nasopharyngitis biopsy samples (n = 55) were obtained from patients in Nanfang Hospital (Southern Medical University, Guangzhou, China) and were frozen in liquid nitrogen for further study. Informed written consent was obtained from each patient. All samples were pathologically confirmed as NPC. The TNM classification was according to the definitions of the seventh edition of the UICC-American Joint Committee on Cancer staging criteria. The research protocols were approved by the Ethics Committee of Nanfang Hospital and registered in Clinical.trials.gov (ID: NCT01171235).

### Cell culture

Human nasopharyngeal carcinoma cell lines such as 5-8 F, 6-10B, CNE1, CNE2, Sune-1, Hone-1 and C666-1 were cultured in RPMI-1640 medium (HyClone, Thermo scientific Inc, China). HEK 293 T cell line (Cell Bank of Chinese Academy of Science in Shanghai, China) was cultured in DMEM/High Glucose medium (HyClone, Thermo scientific Inc, China). The above cell lines were supplemented with 10% fetal bovine serum (FBS, HyClone, Thermo scientific Inc, China). The immortalized nasopharyngeal epithelial cell NP69 (Cancer Research Center, Southern medical university, China) was cultured as the control cell line in Keratinocyte-SFM (GIBICO, Life Technologies corporation, USA).

### RNA isolation, reverse transcription, and quantitative Real-time PCR

Total RNA was extracted from the samples using RNAiso Plus (TAKARA, Shiga, Japan) and reversely transcribed to cDNA using the All-in-One First-Strand cDNA Synthesis kit (GeneCopoeia Inc., USA). Quantitative Real-time PCR (qPCR) was performed using All-in-One^TM^ qPCR Mix (Applied GeneCopoeia Inc., USA) on an ABI 7500HT System. U6 and GAPDH snRNA were used as miRs and miRNA endogenous control respectively. All samples were normalized to the internal control, and the relative expression levels of miR-124 and Foxq1 were calculated using relative quantification assay. Primer sequences for qRT-PCR were displayed in Additional file 6: Table S2.

### Oligo-nucleotide transfection

MiR-124 mimics, miR-Ctrl, Foxq1 siRNAs and Foxq1-Ctrl were synthesized from Gene-pharma (Shanghai, China). RNA oligonucleotides were transfected by lipofectamine 2000 reagent (Invitrogen, Carlsbad, CA, USA).

### Plasmid construction and stable transfection

To obtain stable cell lines to overexpress miR-124, pre-miR-124 was cloned into the pLVTHM lentiviral vector, and the recombinant plasmid was named as lv-miR-124. The lentivial vectors and packing system (psPAX2 and PMD2G) were co-transfected in HEK293T cells by calium phosphate precipitation. Then lentivial virus was collected to infect the 5-8 F and 6-10B cell lines.

The 3′-UTR of Foxq1 was amplified from human genomic DNA and cloned into the psicheck.2 vector (Promega, USA). The mutation of the 3′-UTR of Foxq1 was carried out using site-directed mutagenesis kit (Invitrogen, Carlsbad, CA, USA).

The lentivial viruses to overexpress Foxq1 were purchased from Genechem (Shanghai, China). Then they were used to infect 5-8 F cell lines which stably overexpressed miR-124. The transfected cells were selected by Flow Cytometry.

### Bioinformatics analysis

The probable target genes of miR-124 were predicted using three microRNA target database (PicTar, TargetScan and PITA), and the selected targets gene were validated by RT-qPCR and Western Blot.

### Dual-luciferase assay

Cells were cultured in 24-well plates for Dual-luciferase report system. Cells were co-transfected with 200 ng Wild or Mutant type reporter plasmid and 20 nmol miRNA or anti-miRNA using lipofectamine 2000 reagent (Invitrogen). Cells were harvested and lysed after 24 hours transfection. The firefly and Renilla luciferase activities were measured using the Dual-Glo luciferase reporter assay kit (Promega, Madison, WI, USA).

### Cell proliferation and colony-formation assay

To determine the effect of miR-124 on cell proliferation, cells with stable overexpression of miR-124 were seeded into 96 wells plates at a density of 1 × 10^3^ cells/well with five replicate wells. The effect of miR-124 on cell growth and viability was determined by Cell counting kit 8 assay as described previously [43]. To measure colony-forming activity, cells were counted and seeded into 12-well plates at 100 cells per well. Twelve days after seeding, the numbers of colonies containing more than 50 cells were stained with crystal violet and counted.

### Migration and invasion assays *in vitro*

Transwell migration assay and invasion assay were conducted to determine the functional effects of miR-124 on cell migration and invasion as described previously [14].

### Cell cycle assays

To determine cell cycle distribution, the cells were plated in 6-well plates and transfected with miRNA mimics or siRNA duplexes. After transfection, the cells were harvested, treated with cell cycle detection kit (keygentec Nanjing China) and tested using a FACSCalibur instrument (Becton Dickinson, CA, USA). The data were analyzed using the CellQuest Pro software (BD Biosciences).

### Tumor xenograft model and tumor metastasis assay *in vivo*

Twelve female BALB/C nude mice about four to five-week-old were purchased from Centre of Laboratory Animal of Southern Medical University, and the animal protocol was approved by Institutional Animal Care and Use Committee of Southern Medical University. Twelve nude mice were randomly divided into two groups. 1 × 10^5^ lv-miR-124/5-8 F or lv-miR-Ctrl/5-8 F cells were injected into the dorsal flank of each mouse. Tumor size was measured every other day. Mice were sacrificed and tumors were dissected and weighed. Tumor volumes were calculated as follows: volume = (D × d^2^)/2, where D = the longest diameter and d = the shortest diameter.

For tumor metastasis assay *in vivo*, we used a murine model of NPC metastasis successfully established previously [44]. To establish this model, we inoculated NPC cells into the liver as a single nodule, which would subsequently metastasized to other parts of the liver and the lung. Ten nude mice were randomly divided into two groups. 1 × 10^6^ lv-miR-124/5-8 F or lv-miR-Ctrl/5-8 F cells were injected into the liver of each mouse. The mice were killed and autopsied on day 25, and the incidence of lung or liver metastasis were recorded.

### Western blot

Protein lysates extracted from cell lines were separated by 10% SDS-PAGE, and electrophoretically transferred to PVDF (polyvinylidene difluoride) membrane (Millipore). Then, the membrane was incubated with goat polyclonal antibody against human Foxq1 (WuXi PharmaTech Cayman China) followed by HRP (horseradish peroxidase)-labeled goat-anti mouse IgG (Santa Cruz Biotechnology) and detected by chemiluminescence. Glyceraldehyde-3-phosphate dehydrogenase (GAPDH) was used as a protein-loading control.

### Immunohistochemical analysis

Formalin-fixed, paraffin-embedded tissues of transplanted tumors were sectioned at 4-mm thickness and analyzed for anti-Foxq1 primary antibody (Abcam Ltd, Cambridge, UK), anti- Ki-67 primary antibody, and anti- PCNA primary antibody (Cell Signaling Technology, Inc.USA). Visualization was achieved using the EnVisionþ peroxidase system (Dako). A sample was considered positive if more than 50% of the tumor cells retained nuclear staining, and 5 fields were randomly selected according to semiquantitative scales. The intensity of staining was scored manually: negative (-), weak positive (+), medium positive (++), strong positive (+++). Data recording and analysis by 2 independent experienced pathologists, and only tumor cells were scored.

### Statistical analysis

Statistical analyses were conducted using spss13.0 statistical software. All experiments were performed for three times. The data are shown as the mean ± SEM unless otherwise noted. Two-tailed Student’s *t* test was used for comparison of two independent groups. A one-way ANOVA analysis of variance was used to compare multiple groups. MiR-124 and Foxq1 expressions between tumor and control specimens were analyzed by a Mann–Whitney U test. The relationship between miR-124 and Foxq1 was analyzed using Spearman’s correlation analysis. *P* values of <0.05 were considered statistically significant.

## Electronic supplementary material

Additional file 1: Figure S1: The expression levels of miR-124 in tumor xenograft model and tumor metastasis assay *in vivo.* **A**, The expression levels of Lv-miR-124/5-8F compared with Lv-miR-Ctrl/5-8F in tumorigenesis in murine models. **B**, The expression levels of Lv-miR-124/5-8F compared with Lv-miR-Ctrl/5-8F in tumor metastasis murine models. Statistical analysis was performed using the t-tests. The data represent the mean values of three independent experiments. **, P<0.01. (JPEG 828 KB)

Additional file 2: Table S1: Immunohistochemical analysis of Ki-67 and 662 PCNA expression in transplanted tumors of nude mice. (DOCX 14 KB)

Additional file 3: Figure S2: The ectopic miR-124 induced the expression of Foxq1 by directly targeting its 3′-UTR. **A**, The 27 possible target genes of mir-124 were predicted by bioinformatic analysis. Results of qRT-PCR showed the relative expression of Foxq1 was the most significant down-regulated in lv-miR-124/5-8F cells compared with lv-miR-Ctrl/5-8F. **B**, The mRNA expression level of Foxq1 in lv-miR-124/5-8F cell and lv-miR-124/6-10B cell compared with control cells. **C**, Luciferase reporter assays in HK293T cells, co-transfected of wt/mut 3′-UTR with miRNAs as indicated. Statistical analysis was performed using the t-tests. The data represent the mean values of three independent experiments. *, P<0.05, **, P<0.01. (JPEG 2 MB)

Additional file 4: Figure S3: Down-regulated of Foxq1 inhibited cell proliferation, migration and invasion and the suppression of down-regulated of Foxq1 was consistent with the suppression of the ectopic miR-124. **A**, The mRNA expression levels of Foxq1 in 5-8F cells after tranfected with Foxq1 siRNA and Ctrl-siRNA. **B**, Effect of down-regulated Foxq1 on cell proliferation was measured by CCK-8 assay used 5-8F cell lines. **(C and** **D),** The migratory and invasive cell numbers were detected after tranfected with Foxq1 siRNA and Ctrl-siRNA. **E**, 6-10B cells were transfected with siRNA-Foxq1 or miR-124 mimics. Effect of siRNA-Foxq1 or miR-124 on cell proliferation was measured by CCK-8 assay in 6-10B lines. **(F and** **G)**, The migrated and invasive cell numbers of 6-10B cells. Statistical analysis was performed using the t-tests. The data represent the mean values of three independent experiments. *, P<0.05, **, P<0.01. (JPEG 1 MB)

Additional file 5: Figure S4: Over-expression of Foxq1 could rescue partially the suppression of miR-124 in 6-10B cells. **(A and** **B)**, Effect of up-regulated Foxq1 in lv-miR-124/5-8F cells (lv-Foxq1/lv-miR-124/6-10B) on cell proliferation and tablet cloning ability were measured. **(C and** **D)**, Effect of up-regulated Foxq1 in lv-miR-124/5-8F cells on cell migration and invasion was test. **(E and** **F)**, Stable expression of Foxq1 in lv-miR-124/6-10B cells (lv-Foxq1/lv-miR-124/6-10B) was constructed. Statistical analysis was performed using the t-tests. The data represent the mean values of three independent experiments. **, P<0.01. (JPEG 1 MB)

Additional file 6: Table S2: Primers was used in this study. (DOCX 14 KB)

## Abbreviations

NPC: Nasopharyngeal carcinoma
FOX: Homo sapiens forkhead box
FOXQ1: Homo sapiens forkhead box Q1
MKI67: Homo sapiens marker of proliferation Ki-67
PCNA: Homo sapiens proliferating cell nuclear antigen
TPX2: Homo sapiens TPX2, microtubule-associated
AURKA: Homo sapiens aurora kinase A
VIM: Homo sapiens vimentin
SNAI1: Homo sapiens snail family zinc finger 1
ZEB2: Homo sapiens zinc finger E-box binding homeobox 2
CDH2: Homo sapiens cadherin 2, type 1, N-cadherin.
