# Supplementary figures and images for: MiR-124 suppresses tumor growth and metastasis by targeting Foxq1 in nasopharyngeal carcinoma
Source: Mol Cancer. 2014 Aug 7;13:186. doi: 10.1186/1476-4598-13-186 (PMC4267157; doi:10.1186/1476-4598-13-186)

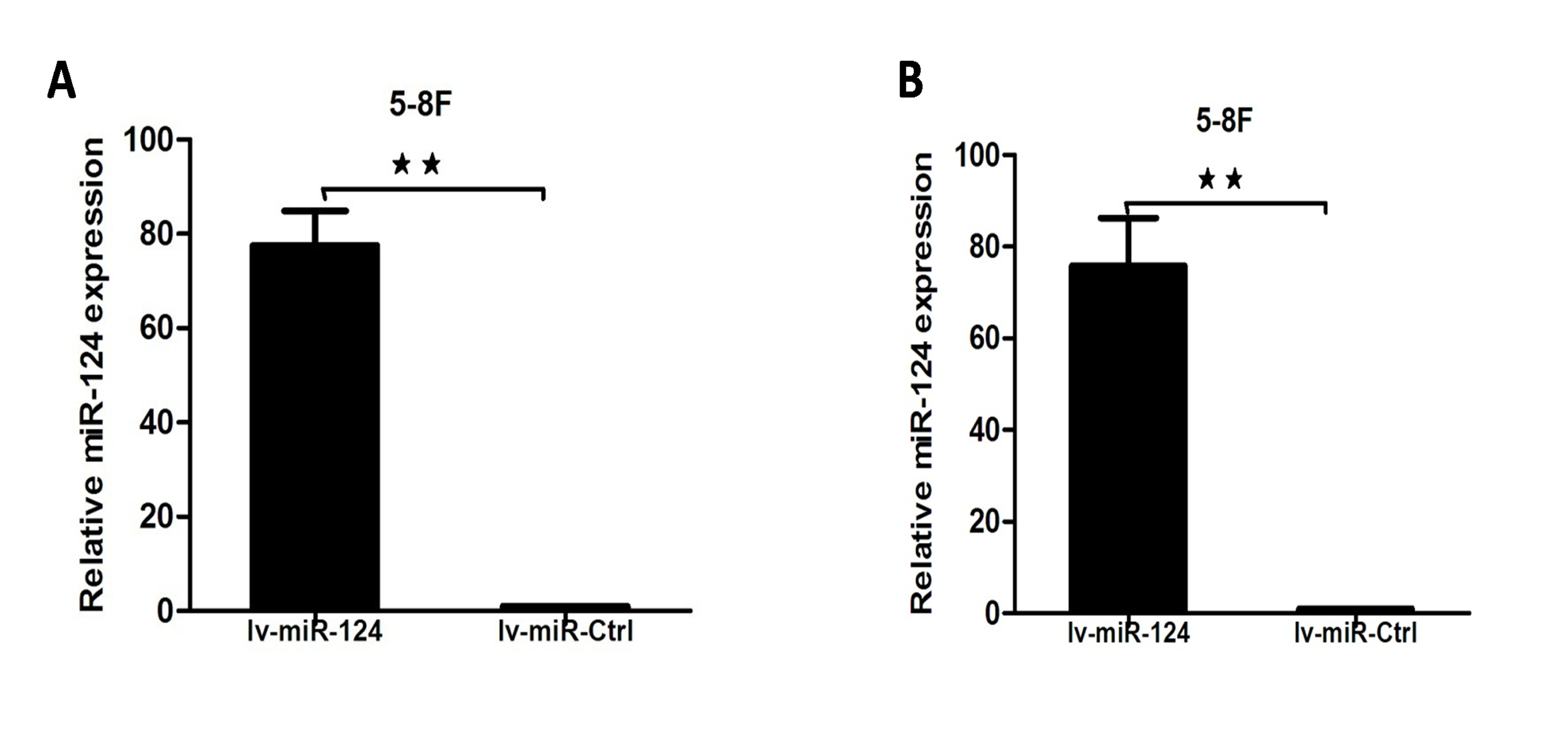

Supplement: Supplementary file 1 — Additional file 1: Figure S1: The expression levels of miR-124 in tumor xenograft model and tumor metastasis assay in vivo. A, The expression levels of Lv-miR-124/5-8F compared with Lv-miR-Ctrl/5-8F in tumorigenesis in murine models. B, The expression levels of Lv-miR-124/5-8F compared with Lv-miR-Ctrl/5-8F in tumor metastasis murine models. Statistical analysis was performed using the t-tests. The data represent the mean values of three independent experiments. **, P<0.01. (JPEG 828 KB) [file 12943_2014_1450_MOESM1_ESM.jpeg]

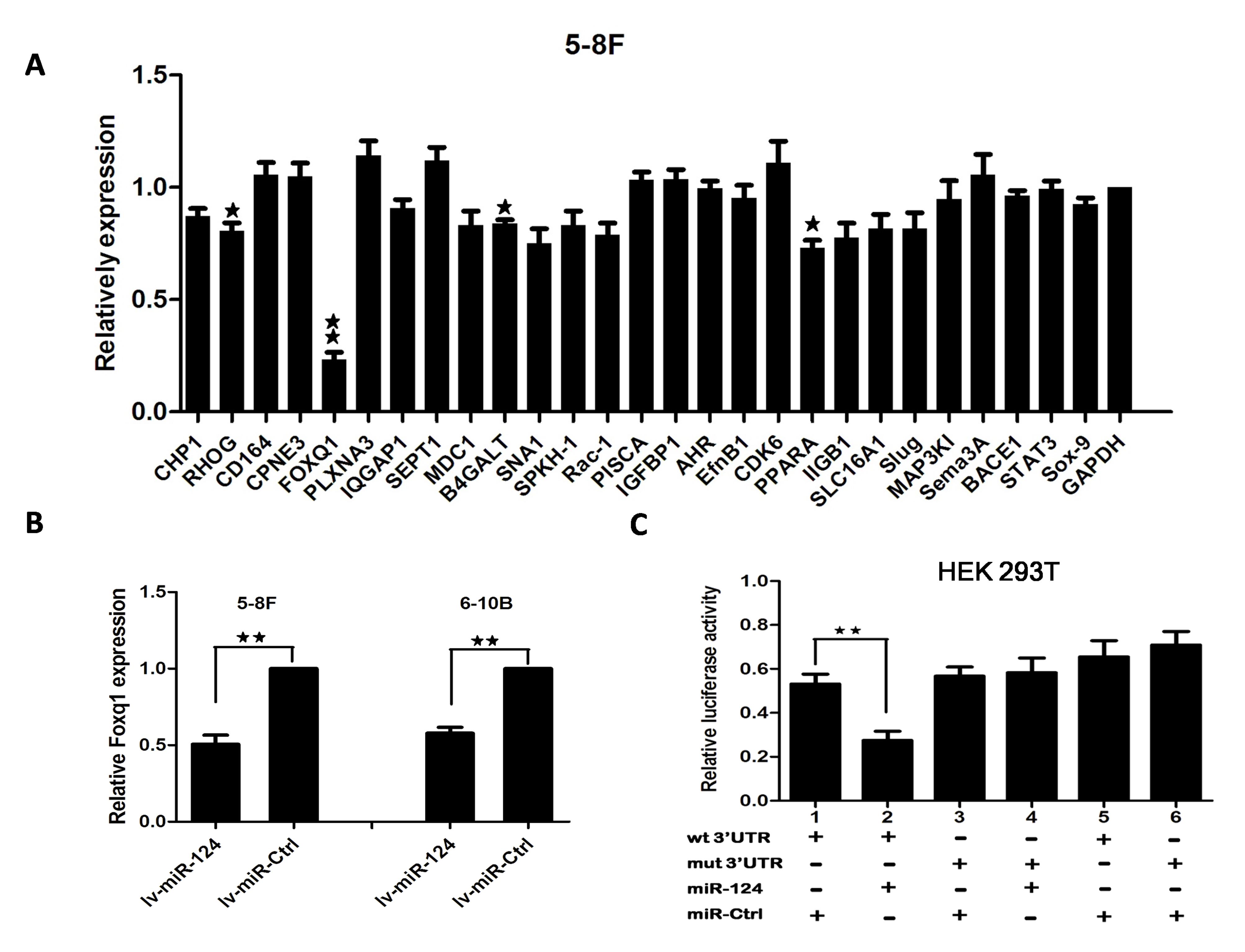

Supplement: Supplementary file 3 — Additional file 3: Figure S2: The ectopic miR-124 induced the expression of Foxq1 by directly targeting its 3′-UTR. A, The 27 possible target genes of mir-124 were predicted by bioinformatic analysis. Results of qRT-PCR showed the relative expression of Foxq1 was the most significant down-regulated in lv-miR-124/5-8F cells compared with lv-miR-Ctrl/5-8F. B, The mRNA expression level of Foxq1 in lv-miR-124/5-8F cell and lv-miR-124/6-10B cell compared with control cells. C, Luciferase reporter assays in HK293T cells, co-transfected of wt/mut 3′-UTR with miRNAs as indicated. Statistical analysis was performed using the t-tests. The data represent the mean values of three independent experiments. *, P<0.05, **, P<0.01. (JPEG 2 MB) [file 12943_2014_1450_MOESM3_ESM.jpeg]

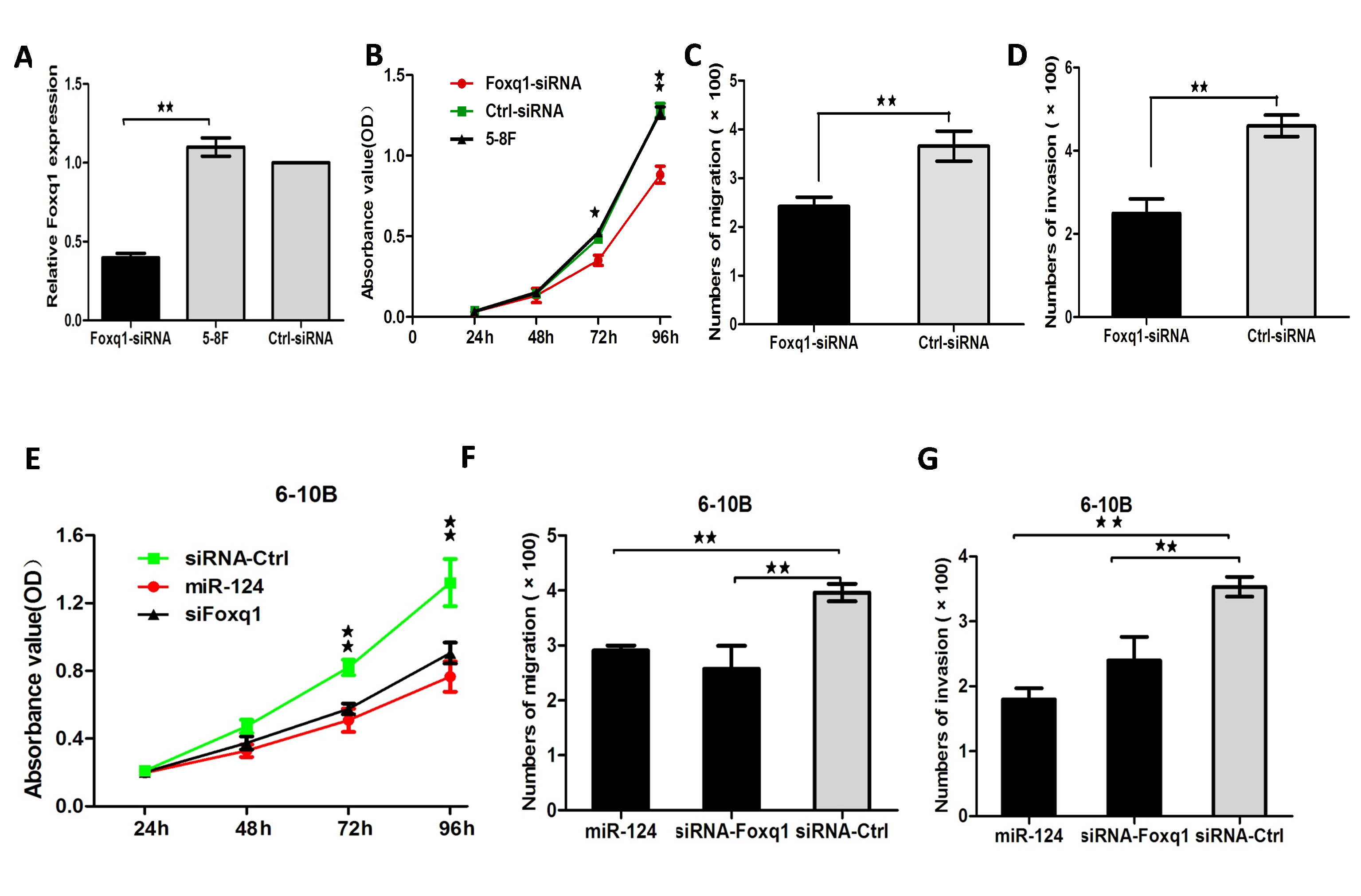

Supplement: Supplementary file 4 — Additional file 4: Figure S3: Down-regulated of Foxq1 inhibited cell proliferation, migration and invasion and the suppression of down-regulated of Foxq1 was consistent with the suppression of the ectopic miR-124. A, The mRNA expression levels of Foxq1 in 5-8F cells after tranfected with Foxq1 siRNA and Ctrl-siRNA. B, Effect of down-regulated Foxq1 on cell proliferation was measured by CCK-8 assay used 5-8F cell lines. (C and D), The migratory and invasive cell numbers were detected after tranfected with Foxq1 siRNA and Ctrl-siRNA. E, 6-10B cells were transfected with siRNA-Foxq1 or miR-124 mimics. Effect of siRNA-Foxq1 or miR-124 on cell proliferation was measured by CCK-8 assay in 6-10B lines. (F and G), The migrated and invasive cell numbers of 6-10B cells. Statistical analysis was performed using the t-tests. The data represent the mean values of three independent experiments. *, P<0.05, **, P<0.01. (JPEG 1 MB) [file 12943_2014_1450_MOESM4_ESM.jpeg]

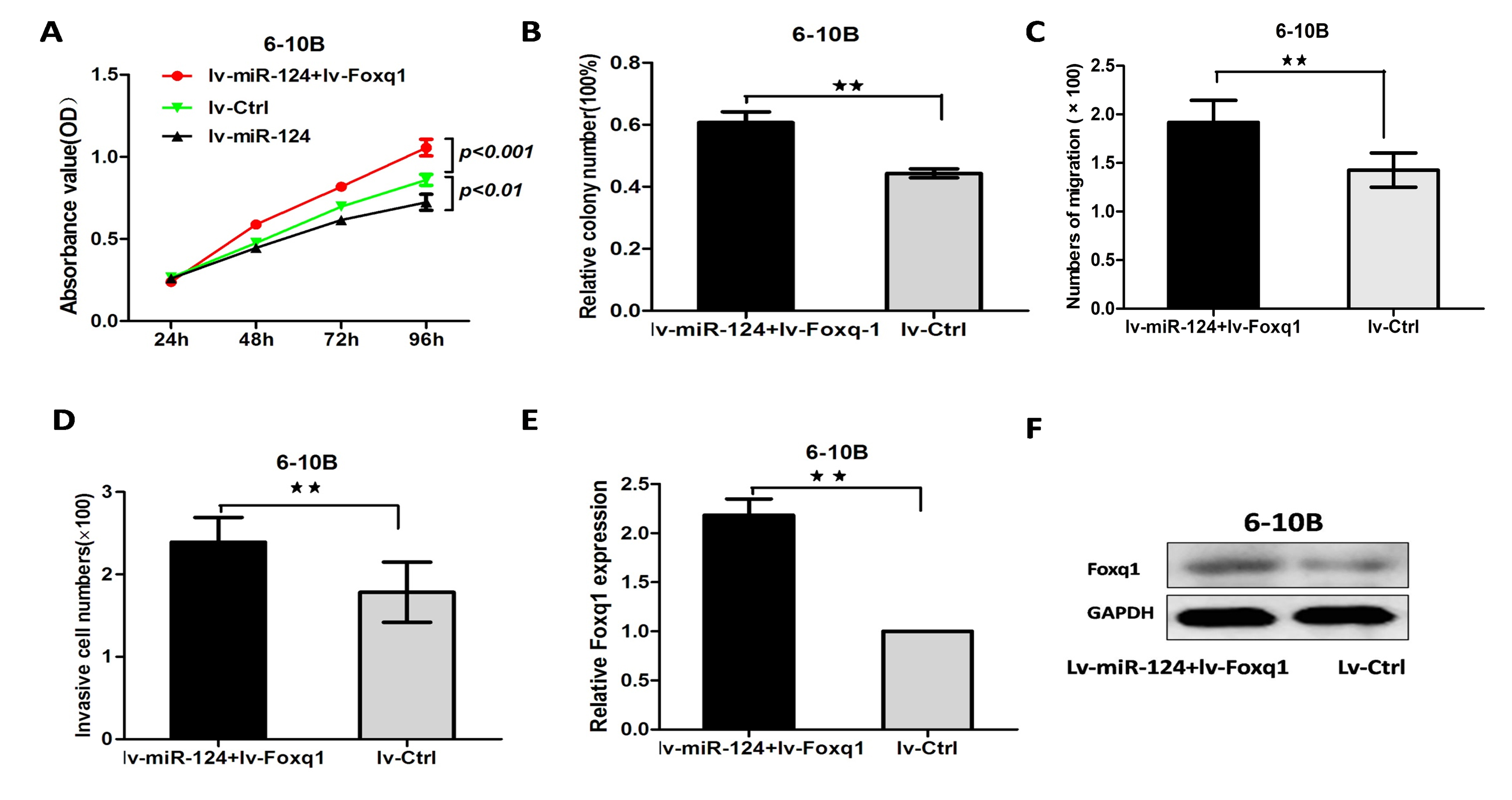

Supplement: Supplementary file 5 — Additional file 5: Figure S4: Over-expression of Foxq1 could rescue partially the suppression of miR-124 in 6-10B cells. (A and B), Effect of up-regulated Foxq1 in lv-miR-124/5-8F cells (lv-Foxq1/lv-miR-124/6-10B) on cell proliferation and tablet cloning ability were measured. (C and D), Effect of up-regulated Foxq1 in lv-miR-124/5-8F cells on cell migration and invasion was test. (E and F), Stable expression of Foxq1 in lv-miR-124/6-10B cells (lv-Foxq1/lv-miR-124/6-10B) was constructed. Statistical analysis was performed using the t-tests. The data represent the mean values of three independent experiments. **, P<0.01. (JPEG 1 MB) [file 12943_2014_1450_MOESM5_ESM.jpeg]
