# Supplementary material for: MiR-124 suppresses tumor growth and metastasis by targeting Foxq1 in nasopharyngeal carcinoma
Source: Mol Cancer. 2014 Aug 7;13:186. doi: 10.1186/1476-4598-13-186 (PMC4267157; doi:10.1186/1476-4598-13-186)
Supplement: Supplementary file 2 — Additional file 2: Table S1: Immunohistochemical analysis of Ki-67 and 662 PCNA expression in transplanted tumors of nude mice. (DOCX 14 KB) [file 12943_2014_1450_MOESM2_ESM.docx]

**Table S1** Immunohistochemical analysis of Ki-67 and PCNA expression in transplanted tumors of nude mice

| Group Fields | | Ki67 | | | | PCNA | | | | |  |
| --- | --- | --- | --- | --- | --- | --- | --- | --- | --- | --- | --- |
|  |  | 0 | 1 | 2 | 3 | *p-*value | 0 | 1 | 2 | 3 | *p-*value |
| lv-miR-124 | 30 | 0 | 20 | 8 | 6 | 0.000 | 0 | 17 | 14 | 3 | 0.000 |
| Lv-miR-con | 30 | 0 | 11 | 20 | 27 |  | 0 | 3 | 30 | 30 |  |

Negative = 0 weak positive= 1 medium positive = 2 strong positive = 3
