# Supplementary material for: MiR-124 suppresses tumor growth and metastasis by targeting Foxq1 in nasopharyngeal carcinoma
Source: Mol Cancer. 2014 Aug 7;13:186. doi: 10.1186/1476-4598-13-186 (PMC4267157; doi:10.1186/1476-4598-13-186)
Supplement: Supplementary file 6 — Additional file 6: Table S2: Primers was used in this study. (DOCX 14 KB) [file 12943_2014_1450_MOESM6_ESM.docx]

**Table S2.** Primers was used in this study

| Gene | Forward sequence | Reverse sequence |
| --- | --- | --- |
| MiR-124 | 5’-TAAGGCACGCGGTGAATGCC-3’ |  |
| Foxq1 | 5’-GCACGCAGCAAGCCATATAC-3’ | 5’-CGCGGAAAAAGGGGAACTTG-3’ |
| Wt 3’-UTR | 5’-ATAAGAATGCGGCCGCGAGGCG’  GGAACGCGG-3’ | 5’-CCGCTCGAGGCAGGCTTCGCA  AAGAAACT-3’ |
| Mut3’-UTR | 5’-TCTCTCCATCAAACAGTGTGCG  AAGCTAAAGCATTT-3 | 5’-AAATGCTTTAGCTTCGCACACTGTTTGATGGAGAGA-3 |
